# Whole genome non-invasive prenatal testing in prenatal screening algorithm: clinical experience from 12,700 pregnancies

**DOI:** 10.1186/s12884-022-04966-8

**Published:** 2022-08-09

**Authors:** Elena E. Baranova, Olesya V. Sagaydak, Alexandra M. Galaktionova, Ekaterina S. Kuznetsova, Madina T. Kaplanova, Maria V. Makarova, Maxim S. Belenikin, Anton S. Olenev, Ekaterina N. Songolova

**Affiliations:** 1 LLC “Evogen”, Moscow, Russian Federation; 2 Federal State Budgetary Educational Institution of Further Professional Education “Russian Medical Academy of Continuous Professional Education” of the Ministry of Healthcare of the Russian Federation, Moscow, Russian Federation; 3 Moscow City Health Department, City clinical hospital №24, Moscow, Russian Federation; 4 Moscow City Health Department, City clinical hospital №67 named after L.A. Vorokhobova, Moscow, Russian Federation

**Keywords:** cfDNA, Non-invasive prenatal testing, NIPT, Prenatal screening, Fetal aneuploidy, Amniocentesis, Trisomy 21, Trisomy 18, Trisomy 13, Rare autosomal trisomies

## Abstract

**Background:**

A fast adoption of a non–invasive prenatal testing (NIPT) in clinical practice is a global tendency last years. Firstly, in Russia according a new regulation it was possible to perform a widescale testing of pregnant women in chromosomal abnormality risk. The aim of the study—to assess efficiency of using NIPT as a second-line first trimester screening test in Moscow.

**Methods:**

Based on the first trimester combined prenatal screening results 12,700 pregnant women were classified as a high-risk (cut-off ≥ 1:100) and an intermediate-risk (cut-off 1:101 – 1:2500) groups followed by whole genome NIPT. Women from high-risk group and those who had positive NIPT results from intermediate-risk group were considered for invasive prenatal diagnostic.

**Results:**

258 (2.0%) samples with positive NIPT results were detected including 126 cases of trisomy 21 (T21), 40 cases of T18, 12 cases of T13, 41 cases of sex chromosome aneuploidies (SCAs) and 39 cases of rare autosomal aneuploidies (RAAs) and significant copy number variations (CNVs). Statistically significant associations (*p* < 0.05) were revealed for fetal fraction (FF) and both for some patient’s (body mass index and weight) and fetus’s (sex and high risk of aneuploidies) characteristics. NIPT showed as a high sensitivity as specificity for common trisomies and SCAs with an overall false positive rate 0.3%.

**Conclusions:**

NIPT demonstrated high sensitivity and specificity. As a second-line screening test it has shown a high efficiency in detecting fetus chromosomal anomalies as well as it could potentially lower the number of invasive procedures in pregnant women.

## Background

Infant mortality is a major medical and social problem, reflecting the quality of the public health system and the future of the country. The main unfavorable factors promoting to high infant mortality are low socioeconomic status of the country, low quality and availability of medical care, severe maternal, fetal or placental conditions, advanced maternity age [1]. The evolution of reproductive technologies over the last few decades has contributed to an advanced maternal age, which is associated with an increased fetal chromosomal anomaly rates and congenital diseases [2]. Among other reasons congenital diseases lead to as much as 20% of all infant deaths [3].

To evaluate and prevent fetus congenital disease, including chromosomal abnormalities, traditional first trimester screening is performed. It includes blood screening combined with an ultrasound examination in the first trimester of pregnancy. Fetus congenital disease can be well diagnosed by ultrasound examinations only from the 11th gestational week. Biochemical blood pregnancy marker risk assessment of chromosomal fetus anomalies is based on the human chorionic gonadotrophin (hCG), the free-β subunit of hCG, and the pregnancy-associated plasma protein (PAPP-A) blood tests. Though widely used biochemical assessment combined with ultrasound is still an indirect evaluation of chromosomal anomalies and results in a false positive rate of up to 5%, leading to increases number of invasive prenatal diagnostic (IPD) procedures [4, 5].

To address these limitations, a new screening method, known as a non-invasive prenatal testing (NIPT), was introduced [6]. The method is based on the massive parallel sequencing of cell-free DNA (cfDNA) fragments derived from a maternal plasma [7]. The sensitivity of NIPT for most common trisomies including trisomies 21 (T21), 18 (T18), and 13 (T13) is high and reaches up to 99, 96 and 91%, respectively [8] with a false-positive results rate as low as 0.08% compared to traditional prenatal screening [9].

There are several types of NIPT – tests targeted on chromosomes of interest (usually 21, 18 and 13) and a whole genome NIPT, that allows to assess all chromosomes and its anomalies. Whole genome NIPT can be used to detect sex chromosome abnormalities and other anomalies, including rare autosomal aneuploidies (RAAs) and significant copy number variations (CNVs), though accuracy of the test for these anomalies is a bit lower due to the fact that RAAs are usually present as a placental mosaicism or a true fetal mosaicism. That could be associated with adverse pregnancy outcomes such as early miscarriage, intrauterine growth restriction, and in-utero fetal demise [10]. In nonmosaic form these chromosomal anomalies usually lead to a fetus death [11].

The American College of Obstetricians and Gynecologists (ACOG) originally suggested the use of NIPT for women previously determined to be in a high-risk group by traditional screening [12]. But it has been shown that the sensitivity and specificity of the test among all pregnant women are similar to those in the high-risk population [13]. Therefore, current international guidelines recommend the use of NIPT for prenatal screening during pregnancy for all women, regardless of the predetermined risk of fetal anomalies [5, 13].

Until recently, in Russia NIPT was primarily a commercially available test used to supplement other screening approaches. On March 13, 2020, NIPT was included as a standard method for prenatal screening as part of a pilot project in Moscow [14]. The large-scale project was run by the Moscow City Healthcare Department with the participation of 23 prenatal care hospitals and one genetic laboratory. Since the beginning, work on national standards and clinical guidelines for NIPT has been ongoing. The aim of the study was to assess the efficiency of NIPT as a second-line first trimester screening test in Moscow. Preliminary results were published before [15]. The project is finished now, and its first results are presented.

## Methods

### Recruitment criteria

**Fig. 1:**
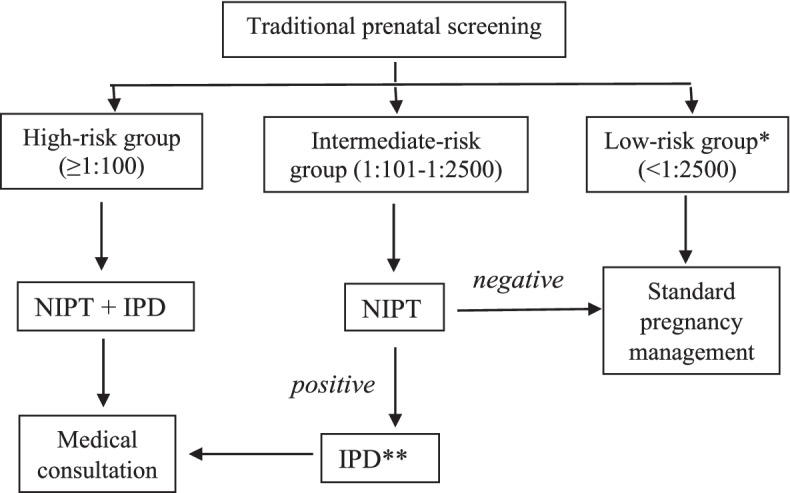
Recruitment criteria for samples that were able to undergo NIPT. *Low-risk group samples were not included into the study. **IPD is obligatory for a high-risk group of pregnant women and is optional for an intermediate-risk group. Abbreviations: NIPT, non–invasive prenatal testing; IPD, invasive prenatal diagnostic

Pregnant women at high-risk of fetus chromosomal anomalies (cut-off ≥ 1:100) after the traditional prenatal screening were referred for genetic counseling, a IPD and a blood sampling for NIPT according the local regulation [14]. Blood test for NIPT was performed in the same day of the IPD (Fig. 1).

Pregnant women at group of risk 1:101–1:2500 were as well offered to perform NIPT. When receiving a positive (high-risk) NIPT result, pregnant women were considered to undergo a genetic consulting and IPD.

### Sample’s collection and blood preparation for sequencing

To perform NIPT 10.0 ml pregnant women peripheral blood samples were collected in STRECK (Cell-Free DNA BCT CE) tubes before IPD. Plasma was separated within 8 h following a double-centrifugation protocol. Tubes were stored temporarily (up to one week) at -20 °C before further processing or stored at – 80 °C for a long-term storage.

### Sequencing

The cfDNA isolation, sample library preparation and DNA sequencing were performed according to manufacturer’s protocols. Each sample was sequenced using a BGISEQ-500 (China) platform. Sequencing reads were trimmed and aligned to a universal unique read set incised from the human reference genome (hg19, NCBI build 37). Combined GC-correction and z-score testing methods were used to identify fetal autosomal aneuploidies. The quality control parameters were as follows: the library concentration was higher than 4 ng/μL; the unique mapped reads number was higher than 6 × 106; the GC content was 38%–42%; and the fetal DNA fraction was higher than 3.5%. The original BGI proprietary software (HALOS NIFTY-2.3.2.1011) was used for a bioinformatic data processing.

### Fetal fraction calculation

The fetus fetal fraction (FF) was calculated using the FF-QuantSC method, that employs neural network model and utilizes differential genomic patterns between fetal and maternal genomes [16].

### NIPT results

The final report included a risk assessment for T21, T18, T13 and SCAs. Whole genome results for RAAs and clinically significant CNVs were included in the report optionally for women who consented to receive that information.

### Invasive prenatal diagnostics

Pregnant women from the high-risk group as well as women with a fetal chromosomal abnormalities risk revealed by NIPT («positive» NIPT results) from the intermediate-risk group were advised to undergo IPD by amniocentesis or chorionic villus sampling (CVS) with a subsequent karyotyping and/or array-based comparative genomic hybridization (aCGH). IPD was also recommended for pregnant women with abnormalities during their second trimester ultrasound examination. CVS or amniocentesis were performed under sterile conditions and an ultrasound guidance in specialized medical hospitals in Moscow.

### Karyotype analysis

For karyotype analysis, 10–20 ml of amniotic fluid was obtained by amniocentesis. The amniotic fluid cells with 4.5 ml medium (RPMI-1640, Paneco, Russia) were cultured in a 37 °C incubator with 5% carbon dioxide. The cells were harvested at 10–12 days. After colchicine treatment for 2 h, the cells were digested using 1:250 trypsin, and incubated with 0.075 M KCl for 30 min. The prefixation, fixation, dropping, baking, and G-band staining were performed next. A total of 100 dividing phases were counted using an all-chromosome image analysis system based on the “An International System for Human Cytogenetic Nomenclature, ISCN2016”.

### aCGH

Agilent SurePrint G3 human aCGH array 8*60 K chips were used for aCGH. DNA from amniotic fluid or chorionic villus after IPD was extracted (New iGENatal Kit, igen biotech, Spain). Agilent CytoGenomics (version 5.0) was used for data analyses. CNVs were classified through Online Mendelian Inheritance in Man (OMIM), Database of Genome Variants (DGV) and Decipher databases. Pathogenic, likely pathogenic, variant of uncertain significance (VUS), likely benign, and benign categories were used for CNVs’ allocation. For VUS aCGH was further performed for parents to verify whether the CNVs were inherited from the parents with a normal phenotype. Inherited CNVs from the parents with a normal phenotype were considered as benign.

### Statistics

Statistical analysis was performed as described earlier [15]. The statistical software package Wizard 2 Version 2.0.4 (250) was used for data analyses.

## Results

**Fig. 2:**
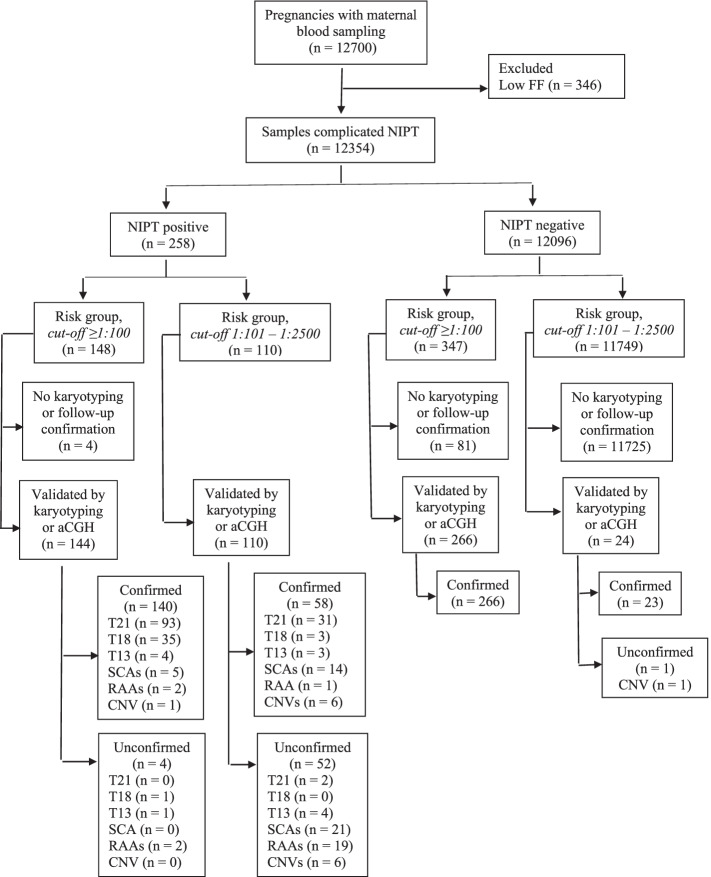
Flowchart of non-invasive prenatal test (NIPT) results and invasive prenatal diagnostics outcomes of pregnant women undergoing screening for aneuploidies between 1 April 2020 and 5 April 2021. Abbreviations: T, trisomy; SCA, sex chromosomal aneuploidy; RAA, rare autosomal aneuploidy; CNV, copy number variation; aCGH, array comparative genomic hybridization

All in all, 12,700 pregnant women blood samples were analyzed during the Apr.1, 2020 till Apr. 5, 2021 period (Fig. 2). The average pregnant women age was 34.4 ± 4.3 years. 53.2% (6756/12700) of pregnant women were in the advanced maternal age (35 and older). The average gestation period was 14 weeks and 1 day ± 1 week and 3 days. 36.7% (4655/12700) of pregnant women had a body mass index (BMI) above 25 kg/m^2^. The majority of pregnancies were spontaneous (96.1%, 12,198/12700) and singleton (98.5%, 12,512/12700) (Table 1).

**Table 1:** Demographic characteristics of women who underwent NIPT and pregnancy characteristics^a^

Maternal characteristics	Samples (*n* = 12,700)
**Age, years**	34.4 (15.0;59.0)
Under 20	65 (0.5)
20–29	2209 (17.5)
30–39	8478 (66.7)
Above 40	1948 (15.3)
**Body mass index (BMI, kg/m**^**2**^**)**	24.5 (14.2;57.6)
< 18.49	624 (4.9)
18.5–24.99	7421 (58.4)
25–29.99	2952 (23.3)
> 30	1703 (13.4)
**Gestation at sampling**	14^+1^ ± 1^+3^ (11^+2^;24^+3^)
11^+0^ to13^+6^ weeks	6202 (48.8)
14^+0^ to 20^+6^ weeks	6464 (50.9)
21^+0^ weeks and above	33 (0.3)
**Type of pregnancy**	
Singleton	12,512 (98.5)
Twins	165 (1.3)
Vanishing twin	23 (0.2)
**Type of conception**	
Spontaneous	12,201 (96.1)
Artificial reproductive technology	499 (3.9)
**Result of prior screening tests**	
High risk (cut-off ≥ 1:100)	502 (3.9)
Intermediate risk (cut-off 1:101 – 1:2500)	12,198 (96.1)

^a^Values are median (range) or n (%)

Blood samples of 502 (3.9%) women in the high-risk group and of 12,198 (96.1%) women in the intermediate-risk group were evaluated. Out of 12,700 samples in 346 (2.7%) cases blood retesting was recommended due to a low FF (below 3.5%).

### NIPT results in high-risk pregnant women

Out of 502 women in the high-risk group (≥ 1:100) 148 (29.5%) were considered positive (high risk) by NIPT, and 347 women were considered negative (low risk) (Fig. 2). IPD results data is available only for 144 cases: 140 case was confirmed by IPD, 4 cases were not confirmed.

The following anomalies were confirmed by IPD in 140 cases: T21 (Down syndrome) in 93 cases, T18 (Edward syndrome) in 35 cases, T13 (Patau syndrome) in 4 cases, X-monosomy (Turner syndrome) in 3 cases, X-chromosome disomy with Y-chromosome monosomy (Klinefelter syndrome) in 1 case and Y-chromosome disomy (XYY syndrome) in 1 case, RAAs in 2 cases (T7 and T22), CNVs in 1 case (del7p14.1p11.2). In 2 cases different chromosomal anomaly was shown by IPD: in 1 case balanced translocation between chromosomes 14 and 22 was shown by IPD, when NIPT showed high risk for T22; in 1 case X triploidy by IPD was shown, when NIPT was positive for T18 and monosomy X.

NIPT results were not confirmed in 1 case for T18, in 1 case for T13 and 2 cases for trisomy of chromosome 16 (Fig. 2).

### NIPT results in intermediate-risk pregnant women

Of the 12,198 cases in the intermediate-risk group (1:101 – 1:2500), 110 (0,9%) cases were considered NIPT positive. IPD results data is available for the group: 58 cases were confirmed by IPD, 52 cases were not confirmed.

The following anomalies were confirmed by IPD in 58 cases: T21 in 31 cases, T18 – in 3 case, T13 in 3 cases, X-chromosome monosomy (Turner syndrome) in 5 cases, X-chromosome disomy with Y-chromosome monosomy (Klinefelter syndrome) in 6 cases, X-chromosome monosomy with Y-chromosome disomy (XYY syndrome) in 2 cases, trisomy of X-chromosome in 1 case, RAA in 1 case (T8), CNVs in 6 cases.

NIPT results were not confirmed in 2 cases of T21, 4 cases of T13, 21 cases of SCAs and 19 cases of RAAs and 6 CNVs.

In the risk group, IPD was performed in 24 patients with negative NIPT due to their abnormal 2nd trimester ultrasound results: congenital malformations or ultrasound markers of fetus chromosomal pathology. According to the IPD results: 23 received a normal karyotype in the fetus, in 1 case a pathology was detected—46,XX,del (18)(p11.2)—a deletion of the short arm of chromosome 18 (false-negative result) (Fig. 2).

### Secondary findings

Thirty-nine cases were considered positive for RAAs or CNVs by NIPT (7 cases in the high-risk group and 32 cases in the intermediate-risk group). The most common were T7 (*n* = 6), T16 (*n* = 4), T8 (*n* = 3) and T22 (*n* = 3). Thirty-seven women (5 cases in the high-risk group and 32 cases in the intermediate-risk group) proceeded with IPD, and chromosomal pathology was confirmed only in 10 cases (Fig. 2). These chromosomal anomalies are rare and are not detectable by widely used NIPT techniques.

### NIPT performance

All in all, the following chromosomal abnormalities have been identified and confirmed: T21 in 124 cases; T18 in 38 cases; T13 in 7 cases; SCAs in 19 cases; RAAs or CNVs – in 10 cases. Comparison of NIPT and IPD results in detection of chromosomal abnormalities is presented in Table 2. Totally, the rate of false-positive results was 0.3%.

**Table 2:** NIPT and IPD results in 12,700 pregnant women

№		Positive results	T21	T18	T13	SCAs	RAAs/CNVs
1	NIPT	258	126	40	12	41	39
2	IPD confirmed	198	124	38	7	19	10
3	IPD not confirmed	56	2	1	5	21	27
4	No data	4	0	1	0	1	2

*Abbreviations*: *IPD* Invasive prenatal diagnosis, *NIPT* Non–invasive prenatal testing, *T* Trisomy, *SCA* Sex chromosomal aneuploidy, *RAA* Rare autosomal aneuploidy, *CNV* Copy number variation

IPD results and/or pregnancy outcomes are available for 9941 women, of whom 9737 had no fetal chromosomal abnormalities and 199 had prenatal and/or postnatal chromosomal abnormalities confirmation. On the basis of the NIPT results and the outcome data available, we calculated the performance of the test in detection of chromosomal anomalies. For T21, T18 and 13, SCAs, RAAs and CNVs the sensitivity was 100%, 100%, 100%, and 92.86%; specificity was 99.50%, 99.15%, 97.47% and 96.88%; positive predictive value (PPV) was 98.26%, 91.67%, 57.14% and 44.83%; negative predictive value (NPV) was 100%, 100%, 100% and 99.80%, respectively (Table 3).

**Table 3:** Summarized data of NIPT performance in detecting trisomies 21, 18/13, SCAs and RAAs/CNVs

Chromosomal anomalies	Sensitivity (% (95% Cl))	Specificity (% (95% Cl))	PPV (% (95% Cl))	NPV (% (95% Cl))
T21	100 (96.79–100)	99.50 (98.20–99.94)	98.26 (93.41–99.56)	100 (99.99–100)
T18/13	100 (91.96–100)	99.15 (97.83–99.77)	91.67 (80.57–96.69)	100 (99.99–100)
SCAs	100 (79.41–100)	97.47 (95.63–98.69)	57.14 (43.27–69.98)	100 (99.99–100)
RAAs/CNVs	92.86 (66.13–99.82)	96.88 (94.97–98.20)	44.83 (32.93–57.35)	99.80 (98.68–99.97)

*Abbreviations*: *PPV* Positive predictive value, *NPV* Negative predictive value, *SCAs* Sex chromosome aneuploidies, *RAAs* Rare autosomal aneuploidies, *CNVs* Copy number variations

To calculate NIPT performance parameters the “true positive” results were defined as positive NIPT results that were confirmed by IPD. The “false positive” results were defined as positive NIPT results for chromosomal anomaly that were shown to be negative by follow‐up IPD. The “true negative” results were defined as negative NIPT results confirmed by karyotyping or aCGH results. The “false negative” results were defined as negative NIPT results with an aneuploidy karyotype confirmed by IPD.

### Association of results with fetal fraction

**Fig. 3:**
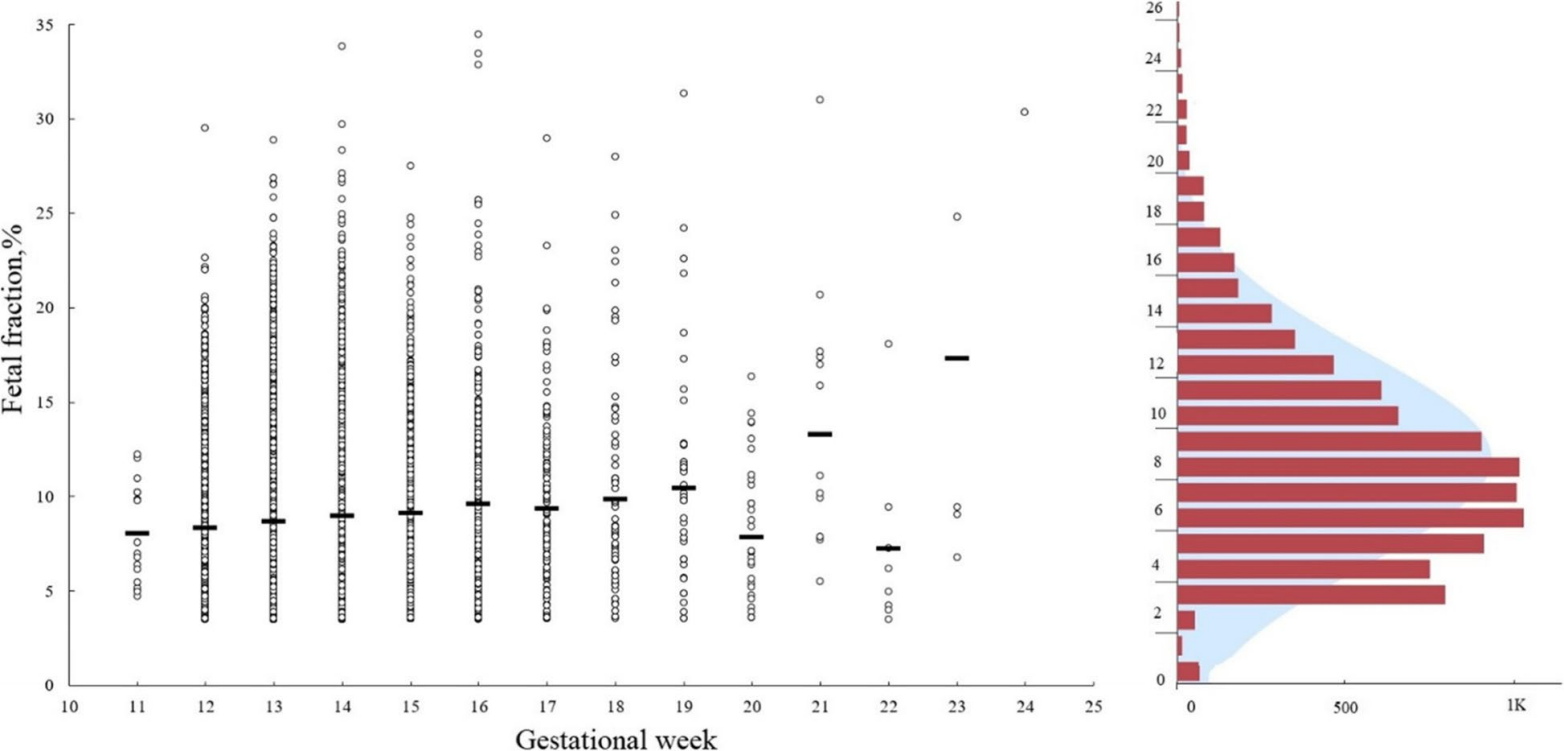
A density distribution of fetal fraction and its relationship with gestational weeks of pregnant women. The average fetal fraction in each week is shown by a black line

It was shown that FF differs between gestational weeks, *p* < 0.001 (Fig. 3).

**Fig. 4:**
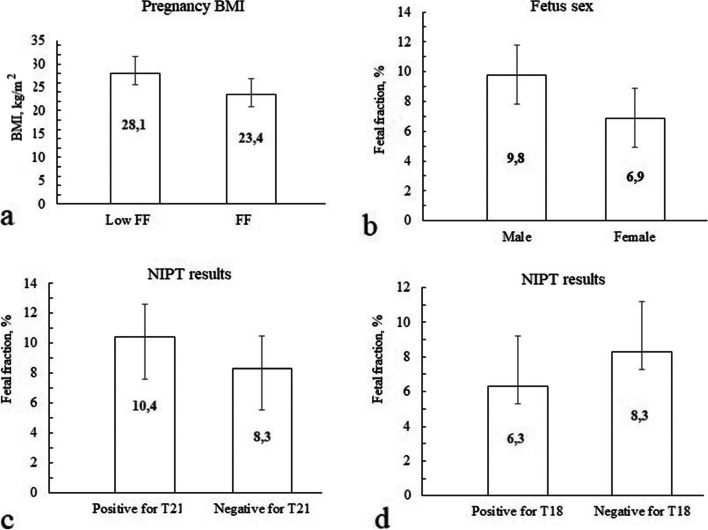
The average level of fetal fraction for patients depending on the BMI (**a**), fetus sex (**b**) and NIPT results: risk of T21 (**c**) and T18 (**d**). Abbreviations: NIPT, non-invasive prenatal testing; T, trisomy; BMI, body mass index.

Also, it was revealed that samples with low FF (< 3.5%) were observed in women with significantly higher weight and BMI in comparison with women with normal FF: 77.5 kg [62.3;91.1] for low FF and 64.0 kg [58.2;74.4] for normal, (*p* < 0.001), BMI – 28.2 kg/m^2^ [23.1;33.6] for low FF and 23.4 kg/m^2^ [21.0;26.8] for normal, *p* < 0.001 (Fig. 4a).

It was also shown, that the FF is significantly higher in women with male fetus, than female: 9.9% [7.4;13.2] and 6.8% [4.9;8.9], *p* < 0.001 (Fig. 4b).

It was also shown, that T21 positive NIPT results were associated with a higher FF, than samples with negative results: 10.4% [7.1;14.4] and 8.3% [6.0;11.2], *p* < 0.001 (Fig. 4c). The cut point for significant difference was 9.0% FF, *p* < 0.001. For T18 it was shown vice versa – T18 positive NIPT results were associated with a lower FF, than samples with negative results: 7.1% [4.4;10.9] and 8.3% [6.0;11.3], *p* = 0.048 (Fig. 4d). The same tendency was not revealed for T13 probably due to a small number of samples.

### NIPT results according to age and mode of conception

We found a significant difference between the age of women with positive and negative NIPT results for T21: 37 yr [33;40] for high T21 risk and 35 yr [31;38] for low risk, *p* = 0.017. Summarizing all the positive NIPT results for all anomalies showed, that these were significantly higher in older women. Mean age of women with a high risk was 37 yr [32;40], and for women with a low risk – 35 yr [31;38], *p* < 0.001.

There was no significant difference in frequency of positive NIPT results in spontaneous and in vitro fertilization pregnancies, *p* = 0.212.

## Discussion

NIPT was first released in Hong Kong in August 2011 and soon after was introduced commercially in the US in October 2011 [17–19]. Afterward, in many countries, multiple companies and their distribution partners offered several NIPT tests to pregnant women, either in a commercial or in a state-regulated setting [20].

The current standard of care for prenatal screening in many high-income countries involves a ultrasound examination combined with biomarker serum screens in the first and/or second trimesters of pregnancy. Despite the fact that in some countries NIPT is performed as a commercial test, or with partial government funding, there is a certain international strategy for introducing NIPT into the structure of prenatal diagnostics. NIPT could be implemented into prenatal testing pipeline in different ways. The most commonly used are as a replacement for serum screening – a first-line test, and as an intermediate step between screening and invasive procedures – a second-line test. The most commonly used implementation model is a combined prenatal screening with the formation of high, intermediate and low risk groups, followed by NIPT in the high and/or intermediate risk group [21]. NIPT as a first-line test is performed to all pregnant women before an expert ultrasound in the first trimester of pregnancy and successfully used in Belgium and the Netherlands [22–25]. NIPT as a second-line test for pregnant women in high or intermediate risk groups determined by the results of combined prenatal screening implemented in some European countries (Germany, Great Britain, France, Italy etc.) [26]. The main advantage of introducing NIPT precisely as a second-line test is economic feasibility.

In Russia the effectiveness of NIPT integration in traditional prenatal screening as a second-line test was shown in the current study. NIPT showed clear accuracy and revealed 37 additional positive (high risk) cases in the intermediate group of pregnant women at risk, compared to traditional prenatal screening. These results included clinically significant CNVs, that were detected only because NIPT was based whole genome sequencing. CNVs are of particular importance because 20 to 30% of congenital diseases are associated with microdeletions and microduplications, which are not detected by traditional prenatal screening and standard cytogenetic studies [27].

The main NIPT advantages are its high sensitivity and specificity for common aneuploidies – T21, T18, T13. Multiple validation studies have reported NIPT high sensitivity (98.6%-100%) and specificity (99.7%-100%) for T21 in different populations [28, 29]. Our results revealed high sensitivity and specificity both for the common trisomies, SCAs and RAAs/CNVs, which is comparable to other studies as well [30, 31].

NIPT shows a very low rate of false-positive and false-negative results compared to traditional prenatal screening results. Several conditions have been known to contribute to false-positive and negative NIPT results: low FF, maternal CNVs and fetal/placental mosaicism are among them [32]. False-negative results are rare for NIPT, with a frequency of only 0.08% [22]. In our study we didn’t have false-negative results for common trisomies, SCAs and RAAs. CNVs false-negative rates was 0.008%.

The false-positive rate for common trisomies in our study reached 0.05%, that is much lower than that reported in other studies [33]. False-positive rate for RAAs and CNVs in our study was 0,09% and 0,04%, respectively. It is assumed that low positive predictive values as well as false-positive rate for CNVs detection are connected with their low frequencies in population.

More over to proven effects there is also one potential effect – decreasing the number of IPD performed in pregnant women. Among 366 women considered to be high risk by traditional prenatal screening, only 105 were confirmed to be high risk by NIPT. That means that 366 women were advised to undergo IPD, although only one third needed these invasive procedures. Currently, the decrease in the number of IPDs is only theoretical, since the regulation of prenatal screening in Russia does not take into account the results of NIPT, and all women at high risk after traditional prenatal screening are considered to undergo IPD.

NIPT has clinical, social and economic benefits. We found social NIPT benefits in its methodology and sample collection. NIPT is safe in blood sampling. Any surgical interventions for are not required. All these can diminish the patient’s anxiety level, which is quite important for pregnant women who may experience hormone-related emotional changes. Moreover, the low false-positive and false-negative rates results reported here and in previous studies [34], suggests that pregnant women can have high confidence in their NIPT results. In our study, we assessed women’s approaches towards NIPT. The results are processing.

Although NIPT is expensive to perform, its economic benefit manifests over an extended period. NIPT can decrease the direct and indirect costs by decreasing budget payments for the maintenance of people with disabilities.

However, despite the obvious advantages of NIPT adoption, there is a downside. The adoption of NIPT in many countries has led to a decrease in IPD procedures, which has had negative consequences, as some authors have proposed [35]. One report has suggested that a decline in IPD procedures causes a downturn in opportunities for physicians to practice the skills needed for IPD procedures, leading to significantly higher miscarriage rates associated with these procedures.

The accuracy of NIPT is affected by numerous factors both biological and technical and include the number of sequencing tags, FF, GC base content, and others. FF is a crucial quality control parameter for NIPT interpretation [36]. Low FF can result in a test failure or a “no call” result. In our study in 2.7% (346/12700) of cases FF was less than 3.5% and a blood sample redraw was required. Any biological factors that increase the maternal contribution and/or reduce the placental contribution may lower the FF [37]: feto-placental – gestational age, crown rump length, mosaicism, fetal aneuploidy, triploidy, multiple pregnancy, and maternal – maternal age, maternal weight, maternal autoimmune disease, low molecular weight heparin, ethnicity, mode of conception [38, 39]. Maternal characteristics such as BMI and gestational age are the main factors that influence FF [40]. Previous data showed that FF below 4% increased with maternal weight from < 1% at 60 kg to > 50% at 160 kg [41]. Therefore, the clinical application of NIPT is limited by low FF of cfDNA in obese women. The rate of increase in FF is not constant across gestational age. From 10–12.5 weeks, 12.5–20 weeks, and > 20 weeks, the FF increases at rates of 0.44%, 0.083%, and 0.821% per week, respectively [42]. Waiting for a later gestational age and repeating blood sampling is not a reliable approach to overcome the low FF in subjects with higher BMIs and earlier gestational ages [43].

In our study, we analyzed the influence of some available parameters on FF and observed no significant differences between FF and maternal age, gestational age, mode of conception and type of pregnancy. However, we noticed a statistically significant decrease in FF with increased BMI and maternal weight.

In our study it was also shown, that higher FF was more common for male fetuses and for fetuses with high risk for T21, lower FF – for fetuses with high risk for T18. The same was also published in some other studies, showing that euploid male fetus pregnancies with high risk of T21 had higher FF [44]. For T18, T13 and monosomy X, vice versa other studies has shown lower FF. Higher FF in fetuses with T21 may be one of the reasons the test performance is better for T21 than for T18 and T13. In our study the cut-off for high T21 risk was 9.0% FF. We didn’t find any significant difference in FF for T13 and monosomy X, that is probably due to low incidence yet.

Pregnant women aged over 35 years are usually categorized as advanced maternal age [42]. It is reported that advanced maternal age is associated with various pregnancy complications, including infant chromosomal anomalies. It is known that such chromosomal abnormalities as T21, T18, T13, triple X syndrome, and XYY syndrome have a close association with maternal age [7]. However, pathogenic chromosomal deletions and duplications also occur de novo, and the risk of microdeletions and microduplications is the same for all pregnancies regardless of maternal age [45]. In our study, we detected a significantly higher risk of the genetic abnormalities in women aged 39 and older.

Some studies have also reported that even when the NIPT result was negative, many other chromosomal anomalies could be detected by other technical methods [46]. The major types of missed fetal abnormalities include structural (balanced or unbalanced) rearrangements, mosaic and triploidies [47]. Chena et al. declare that 12.4% of fetal chromosomal abnormalities will be missed if NIPT completely replaces IPD in advanced aged pregnant women [46]. In 2020 ACOG proposed prenatal screening for aneuploidy for all pregnant women, regardless of age or baseline risk factors [48], but NIPT cannot completely replace IPD in advanced maternal aged women.

## Conclusion

NIPT as a second-line test in Moscow, Russia have shown its effectiveness. The major advantage of NIPT was safety, detection of additional chromosomal anomalies and reduction in false-positive rates. Moreover, our findings suggest that NIPT merits serious consideration as a primary screening method for fetal autosomal aneuploidy. NIPT should be recommended for all pregnant women in risk groups, but using it as a first-tier screening and diagnostic tool requires further study.

## Limitations of the present study

Main limitations are lack of data due to women refused to undergo IPD and lack of information about pregnancies outcomes.

## Data Availability

The datasets generated during and analyzed during the current study are not publicly available due to prohibition of sending raw sequencing data to foreign repositories but are available from the corresponding author on reasonable request.

## Abbreviations

aCGH: Array-based comparative genomic hybridization
ACOG: American College of Obstetricians and Gynecologists
BMI: Body mass index
cfDNA: Cell-free DNA
CNVs: Copy umber variants
CVS: Chorionic villus sampling
DNA: Deoxyribonucleic acid
FF: Fetal fraction
FN: False negative
FP: False positive
GAPDH: Glyceraldehyde-3-phosphate dehydrogenase
hCG: Human chorionic gonadotrophin
IPD: Invasive prenatal diagnosis
NIPT: Non–invasive prenatal testing
NPV: Negative predictive value
PAPP-A: Pregnancy-associated plasma protein A
PPV: Positive predictive value
RAAs: Rare autosomal aneuploidies
T: Trisomy
TP: True positive
TN: True negative
SCA: Sex chromosomal aneuploidy
Yr: Years

## References

[1] MacDorman MF (2011). Race and ethnic disparities in fetal mortality, preterm birth, and infant mortality in the United States: an overview. Semin Perinatol.

[2] Baranov AA, Namazova-Baranova LS, Belyaeva IA, Bombardirova EP, Smirnov IE (2015). Medical and social problems of assisted reproductive technologies from the perspective of pediatrics. Vestn Ross Akad Med Nauk.

[3] Baranov AA, Namazova-Baranova LS, Albitskiy V, Terletskaya RN (2017). Tendencies of infantile and child mortality in the conditions of implementation of the modern strategy of development of health care of the Russian Federation. Vestnic RAMN.

[4] Sukhikh GT, Karetnikova NA, Baranova EE, Shubina ES, Korostin DO, Evdokimov AN (2016). Noninvasive prenatal diagnosis of aneuploidies by high-throughput sequencing (NGS) in a group of high-risk women. Obstet Gynecol (Moscow).

[5] Gregg AR, Skotko BG, Benkendorf JL, Monaghan KG, Bajaj K, Best RG (2016). Noninvasive prenatal screening for fetal aneuploidy, 2016 update: a position statement of the American College of Medical Genetics and Genomics. Genet Med.

[6] Benn P, Cuckle H, Pergament E (2013). Non-invasive prenatal testing for aneuploidy: current status and future prospects. Ultrasound Obstet Gynecol.

[7] Pös O, Budiš J, Szemes T (2019). Recent trends in prenatal genetic screening and testing. F1000Res.

[8] Taylor-Phillips S, Freeman K, Geppert J, Agbebiyi A, Uthman OA, Madan J, Clarke A, Quenby S, Clarke A (2016). Accuracy of non-invasive prenatal testing using cell-free DNA for detection of Down, Edwards and Patau syndromes: a systematic review and meta-analysis. BMJ Open.

[9] Mackie FL, Hemming K, Allen S, Morris RK, Kilby MD (2017). The accuracy of cell-free fetal DNA based non-invasive prenatal testing in singleton pregnancies: a systematic review and bivariate meta-analysis. BJOG.

[10] Neofytou M (2020). Predicting fetoplacental mosaicism during cfDNA-based NIPT. Curr Opin Obstet Gynecol.

[11] Pertile MD, PageChristiaens L, Klein H-G (2018). Chapter 7: Genome-wide cell-free DNA-based prenatal testing for rare autosomal trisomies and subchromosomal abnormalities. Noninvasive prenatal testing (NIPT) [Internet].

[12] Noninvasive Prenatal Testing for Fetal Aneuploidy. Available from: http://www.acog.org/Resources-And-Publications/Committee-Opinions/Committee-on-Genetics/Noninvasive-Prenatal-Testing-for-Fetal-Aneuploidy. Accessed July 28, 2014.

[13] Porreco RP, Garite TJ, Maurel K, Marusiak B, Ehrich M, van den Boom D (2014). Noninvasive prenatal screening for fetal trisomies 21, 18, 13 and the common sex chromosome aneuploidies from maternal blood using massively parallel genomic sequencing of DNA. Am J Obstet Gynecol.

[14] Prikaz Minzdrava goroda Moscow № 199 ot 13.03.2020 «Ob organizatsii provedeniya neinvasivnogo prenatalnogo testa v gorode». https://www.mos.ru/dzdrav/documents/department-acts/view/237308220/.

[15] Olenev AS, Baranova EE, Sagaidak OV, Galaktionova AM, Kuznetsova ES, Kaplanova MT (2021). Adoption of a non-invasive prenatal test (NIPT) in prenatal screening in Moscow: first results. Rus Open Med J.

[16] Yuan Y, Chai X, Liu N, Gu B, Li S, Gao Y (2020). FF-QuantSC: accurate quantification of fetal fraction by a neural network model. Mol Genet Genomic Med.

[17] Lau TK, Chan MK, Lo PS (2012). Clinical utility of noninvasive fetal trisomy (NIFTY) test – early experience. J Matern Fetal Neonatal Med.

[18] Agarwal A, Sayres LC, Cho MK, Cook-Deegan R, Chandrasekharan S (2013). Commercial landscape of noninvasive prenatal testing in the United States. Prenat Diagn.

[19] Chandrasekharan S, Minnear MA, Hung A, Allyse M. Noninvasive prenatal testing goes global. Sci Transl Med. 2014;6(231):231fs15. 10.1126/scitranslmed.3008704 PMC4112725 24718856

[20] Bianchi DW, Chiu RWK (2018). Sequencing of circulating cell-free DNA during pregnancy. N Engl J Med.

[21] Gil MM, Revello R, Poon LC, Akolekar R (2016). Clinical implementation of routine screening for fetal trisomies in the UK NHS: cell-free DNA test contingent on results from first-trimester combined test. Ultrasound Obstet Gynecol.

[22] Bianchi DW, Wilkins-Haug L (2014). Integration of noninvasive DNA testing for aneuploidy into prenatal care: what has happened since the rubber met the road?. Clin Chem.

[23] van Schendel RV, van El CG, Pajkrt E, Henneman L, Cornel MC (2017). Implementing non-invasive prenatal testing for aneuploidy in a national healthcare system: global challenges and national solutions. BMC Health Serv Res.

[24] Karuna RM, van der M, Sistermans EA, Macville MVE, Stevens SJC, Bax CJ (2019). TRIDENT-2: National implementation of genome-wide non-invasive prenatal testing as a first-tier screening test in the Netherlands. AJHG.

[25] Neyt M, Hulstaert F, Gyselaers W (2014). Introducing the non-invasive prenatal test for trisomy 21 in Belgium: a cost-consequences analysis. BMJ Open.

[26] UK National Screening Committee non-invasive prenatal testing (NIPT) recommendation. January 2016; Department of Health and Social Care. The UK Strategy for Rare Diseases. 2020 update to the Implementation Plan for England. Published: 26 February 2020. https://www.gov.uk/government/publications/uk-strategy-for-rarediseases-2020-update-to-the-implementation-plan-for-england.

[27] Hongtai L, Gao Y, Hu Z, Lin L, Yin X, Wang J (2016). Performance evaluation of NIPT in detection of chromosomal copy number variants using low-coverage whole-genome sequencing of plasma DNA. PLoS ONE.

[28] Liang D, Lin Y, Qiao F, Li H, Wang Y, Zhang J (2018). Perinatal outcomes following cell-free DNA screening in >32 000 women: clinical follow-up data from a single tertiary center and has relative lower sensitivities and specificities for T18, T13 and SCAs. Prenat Diagn.

[29] Petersen AK, Cheung SW, Smith JL, Bi W, Ward PA, Peacock S (2017). Positive predictive value estimates for cell-free noninvasive prenatal screening from data of a large referral genetic diagnostic laboratory. Am J Obstet Gynecol.

[30] Luo Y, Hu H, Jiang L, Ma Y, Zhang R, Xu J (2000). A retrospective analysis the clinic data and follow-up of non-invasive prenatal test in detection of fetal chromosomal aneuploidy in more than 40,000 cases in a single prenatal diagnosis center. Eur J Med Genet.

[31] Ramdaney A, Hoskovec J, Harkenrider J, Soto E, Murphy L (2018). Clinical experience with sex chromosome aneuploidies detected by noninvasive prenatal testing (NIPT): Accuracy and patient decision-making. Prenat Diagn.

[32] Samura O, Okamoto A (2020). Causes of aberrant non-invasive prenatal testing for aneuploidy: a systematic review. Taiwan J Obstet Gyn.

[33] Wang Y, Li S, Wang W, Dong Y, Zhang M, Wang X, Yin C (2020). Cell-free DNA screening for sex chromosome aneuploidies by non-invasive prenatal testing in maternal plasma. Mol Cytogenet.

[34] Beaudet AL (2016). Using fetal cells for prenatal diagnosis: History and recent progress. Am J Med Genet C Semin Med Genet.

[35] Hui L, Tabor A, Walker SP, Kilby MD (2016). How to safeguard competency and training in invasive prenatal diagnosis: ‘the elephant in the room’. Ultrasound Obstet Gynecol.

[36] Kinnings SL, Geis JA, Almasri E, Wang H, Guan X, McCullough RM (2015). Factors affecting levels of circulating cell-free fetal DNA in maternal plasma and their implications for noninvasive prenatal testing. Prenat Diagn.

[37] Hui L, Bianchi DW (2020). Fetal fraction and noninvasive prenatal testing: what clinicians need to know. Prenat Diagn.

[38] Lau TK, Zhao L, Yi X, Yin Y, Wang W (2015). Noninvasive prenatal testing for trisomies 21, 18 and 13: clinical experience from 146,958 pregnancies. Ultrasound Obstet Gynecol.

[39] Livergood MC, LeChien KA, Trudell AS (2017). Obesity and cell-free DNA “no calls”: is there an optimal gestational age at time of sampling?. Am J Obstet Gynecol.

[40] Qiao L, Zhang Q, Liang Y, Gao A, Ding Y, Zhao N (2019). Sequencing of short cfDNA fragments in NIPT improves fetal fraction with higher maternal BMI and early gestational age. Am J Transl Res..

[41] Ashoor G, Syngelaki A, Poon LC, Rezende JC, Nicolaides KH (2013). Fetal fraction in maternal plasma cell-free DNA at 11–13 weeks’ gestation: relation to maternal and fetal characteristics. Ultrasound Obstet Gynecol.

[42] Ahkam GK, Abdurrahman Hİ, Emrah B, Suriye Ö, Adnan B (2019). Effect of advanced maternal age on pregnancy outcomes: a single-centre data from a tertiary healthcare hospital. J Obstet Gynaecol.

[43] Qiao L, Zhang Q, Liang Y, Gao A, Ding Y, Zhao N (2019). Sequencing of short cfDNA fragments in NIPT improves fetal fraction with higher maternal BMI and early gestational age. Am J Transl Res..

[44] Wang E, Batey A, Struble C, Musci T, Song K, Oliphant A (2013). Gestational age and maternal weight effects on fetal cell-free DNA in maternal plasma. Prenat Diagn.

[45] Goldenberg P (2018). An update on common chromosome microdeletion and microduplication syndromes. Pediatr Ann.

[46] Chena Y-P, Heb Z-Q, Shia Ye, Zhoua Q, Caia Z-M, Bin Yu (2018). Not all chromosome aberrations can be detected by NIPT in women at advanced maternal age: a multicenter retrospective study. Clin Chim Acta.

[47] Zhu Y, Shiming Lu, Bian X, Wang He, Zhu B, Wang H (2016). A multicenter study of fetal chromosomal abnormalities in Chinese women of advanced maternal age. Taiwan J Obstet Gynecol.

[48] Palomaki GE, Kloza EM (2018). Prenatal cell-free DNA screening test failures: a systematic review of failure rates, risks of down syndrome, and impact of repeat testing. Genet Med.

